# Plasmonic Nanoprism Distributions to Promote Enhanced and Uniform Energy Deposition in Passive and Active Targets

**DOI:** 10.3390/nano15231801

**Published:** 2025-11-29

**Authors:** Dávid Vass, Emese Tóth, András Szenes, Balázs Bánhelyi, István Papp, Tamás Sándor Biró, László Pál Csernai, Norbert Kroó, Mária Csete

**Affiliations:** 1 Department of Optics and Quantum Electronics, University of Szeged, 6720 Szeged, Hungary; 2 Wigner Research Centre for Physics, 1121 Budapest, Hungary; 3 Department of Computational Optimization, University of Szeged, 6720 Szeged, Hungary; 4 Department of Physics and Technology, University of Bergen, 5007 Bergen, Norway; 5 Frankfurt Institute for Advanced Studies, 60438 Frankfurt am Main, Germany

**Keywords:** fusion, nanoresonator distribution, near-field amplification, uniform energy deposition, numerical optimization

## Abstract

Passive and active targets, both implanted with gold nanoprisms, were designed to achieve enhanced, uniform power absorption during two-sided illumination with short laser pulses. The capabilities of uniform, single-peaked Gaussian and adjusted nanoresonator number density distributions were compared. The average local **E**-field inside the gain medium and at the nanoprism surface was mapped as a function of the pump **E**-field strength and dye concentration, and the optimal parameters were selected based on the achievable local **E**-field. A comparative study was performed on passive and active targets to determine the most favorable distribution type and to consider the advantages of dye doping. The adjusted distribution is proposed for both passive and active targets. Dye doping is advantageous in all distributions as it results in decreasing the minimal standard deviation of the near-field enhancement (*NFE*), the delay of the minimal standard deviation in the power loss and deposited energy, and the standard deviation of the *NFE*, while increasing the *FOM* of the *NFE* in the uniform and adjusted distributions. Dye doping allows for decreasing the delay of the minimal standard deviation in the *NFE*, increasing the mean *NFE*, and decreasing the standard deviation of the power loss and deposited energy in the uniform, Gaussian, and adjusted distribution, respectively.

## 1. Introduction

The control of nuclear reactions could address the energy demand; however, sustainable technology to achieve stable fusion has not yet been developed [1]. Various approaches in nanophotonics are focused on enhancing charged particle density [2], accelerating them [3,4], extending the cut-off energy of crucial phenomena [5,6], and improving the conversion efficiency [7].

Most of these approaches rely on boosting the near-field through better confinement and enhancement attainable via various nanophotonic resonators. Large and confined near-fields can be achieved through plasmonic nanoparticles (NPs) of different types, due to their localized surface plasmon resonance (LSPR). The **E**-field enhancement is achievable via plasmonic nanoantennas, and their patterns can reach several orders of magnitude [8,9]. The degree and distribution of the **E**-field enhancement originating from LSPR can be tuned by varying the plasmonic nanoresonator parameters, including their shape, size, composition, as well as the embedding environment [10]. LSPRs can be excited on asymmetric nanoantennas, e.g., nanoprisms, which offer the specific advantage of extreme **E**-field localization in a single hot spot [11]. In the case of individual triangular nanoantennas, efficient excitation of LSPR requires **E**-field oscillation along their long axis, allowing for the strongest **E**-field confinement at the smallest radius of curvature [12].

An almost uniform size distribution of nanoprisms can be achieved through chemical procedures or combined laser and colloid-sphere lithographies [11]. Moreover, ordered patterns of uniform and oriented nanoprisms show a promise of enhancing the **E**-field via surface lattice resonances (SLR) that can be excited when the period is comparable to the wavelength in the specific embedding medium. Such patterns of nanoprisms with controlled location and orientation can be fabricated using nanosphere lithography [13].

Plasmonic nanoparticles (NPs) can be used in a wide range of multidisciplinary applications. As drug delivery systems, these NPs offer competitive methods for cancer treatment by increasing the therapy effectiveness and facilitating the overcoming of various challenges, such as drug resistance [14]. They are also applicable in nanoparticle-based bone tissue engineering, by contributing to increased bone regeneration efficacy [15]. Plasmonic nanoparticles can serve as anatomic and molecular imaging markers as well, due to their small size and high surface-to-volume ratio. Moreover, they offer stable and intense imaging signals, with multimodal and multiplexing capabilities [16].

Due to the large and confined local **E**-fields, nanoparticles can enhance the spontaneous emission of nearby emitters [17]. Accordingly, plasmonic nanoparticles can be used to increase the luminescence of dye molecules; moreover, plasmon-enhanced lasing can also be achieved [18,19]. Quantum dots (QDs) can also effectively couple with metal nanoprisms, in the case of proper geometry tuning. When the LSPR overlaps with the photoluminescence spectrum of the QDs, the emission intensity can be increased, while the lifetime can be decreased according to the Purcell effect [20]. Plasmon-enhanced emission phenomena offer a tool to further boost the **E**-field strength.

In addition to individual nanoresonators, various multitudes of nanoparticles were also studied. Random lasing can be achieved with randomly positioned resonant scatterers inside an optical gain medium. In these systems, the lasing properties are determined by considering the interplay between the gain medium and the scattering centers [21]. Random lasing action can be considerably enhanced with metal nanoparticles, as the scattering cross-section is increased compared to the geometrical cross-section due to the surface plasmon resonance, while the gain volume is decreased due to the **E**-field confinement. The large localized **E**-field in nanoresonator-integrated media ensures control over both absorption and emission phenomena, leading to a considerable fluorescence enhancement [22]. In gain media seeded with nanoprisms, multiple emission spikes appear, and the lasing threshold decreases compared to media without nanoresonators. Pronounced full-width-at-half maximum (*FWHM*) narrowing can be achieved in the presence of oriented nanoparticles due to the coherent feedback, and the threshold is significantly decreased due to the increased local **E**-field and corresponding local pump fluence [23]. Experimental evidence of random lasing action was demonstrated using nanoprisms embedded into a substrate. 

The emission wavelength can be pronouncedly blue-shifted compared to the standard pulling effect due to the strain stemming from the bending of the polymer target, allowing for tunable lasing emission [24]. Plasmonic nanoparticles can be used for various light-controlled energy deposition purposes [25,26,27].

In our previous study, it was shown that by optimizing the distribution of the NPs along an extended target, uniform energy deposition can be achieved, which is crucial in fusion applications [28]. The possibility of balancing the deposited energy along an extended target was demonstrated using core–shells and nanorods, both in passive and active media [28,29]. However, when extended targets are illuminated by short laser pulses, the time evolution of the induced phenomena is governed by the laser pulse shape. Accordingly, it was demonstrated that to achieve uniformly distributed near-field enhancement, double-sided illumination is advantageous [30]. This is an important tool to avoid instabilities that can arise in the case of three-fold, namely, spectral-spatial-temporal, laser intensity confinement. This work explores a physical regime of lower-intensity pulses—well below the breakdown threshold—that can already yield considerable and spatially controllable energy deposition, when mediated by local plasmonic near-field enhancement. The goal is not to replicate inertial confinement fusion (ICF) conditions, but to investigate whether plasmonic nanoprisms can act as local energy-transfer resonators, concentrating the incident optical field into nanoscale hot spots and thereby enabling locally more efficient and simultaneously more uniform absorption across micrometer-scale targets under moderate pulse energies.

In this study, a theoretical investigation is performed based on the concept that the plasmonic nanoprisms can be advantageous in achieving larger near-field enhancement and power loss along the target. It is shown that by modifying the nanoprism number density distribution, uniform power absorption, near-field enhancement, and energy deposition can be achieved. The advantages of the added dye were also analyzed in the case of three different nanoresonator distributions. Although the concept of tailoring nanoresonator distributions for uniform energy deposition has already been introduced in our previous works on core–shell nanoparticles and nanorods [28,29], the present study provides substantial methodological advances. First, the application of asymmetric triangular nanoprisms fundamentally modifies the achievable values and spatial distribution of the near-field enhancement and the characteristics of the optimal number density distributions via intermediate orientation-dependent plasmon excitation and single hot spot field localization phenomena. Second, a two-component optimization was realized, in which both the nanoprism number density and dye concentration distributions are adjusted. Finally, the comparative time-dependent analysis of passive and active targets is extended. These advances establish that the present work is an extension of previous distribution-optimization studies, which allows for uncovering novel physical phenomena.

## 2. Materials and Methods

Steady-state and time-domain computations were realized using the RF module of COMSOL Multiphysics 6.2 (COMSOL Inc, Stockholm, Sweden). Models, solving Maxwell’s equations in the frequency and time domains, were created. Locally refined meshes were constructed around the nanoprisms to resolve the high-gradient electromagnetic fields near the sharp tips. Special attention was devoted to the accurate computation of the near-field distribution, as these localized fields govern both the spatial uniformity and magnitude of absorption. In COMSOL Multiphysics, the near-field enhancement (*NFE* = *E*_*local*_/*E*_0_) was obtained by directly extracting the local electric-field amplitude (*E_local_*) from the complex field solution and comparing it to the reference field in an identical nanotriangle-free (and passive) reference target (*E*_0_). This approach ensures self-consistent, high-resolution evaluation of all near-field quantities within the passive and active targets.

**Figure 1:**
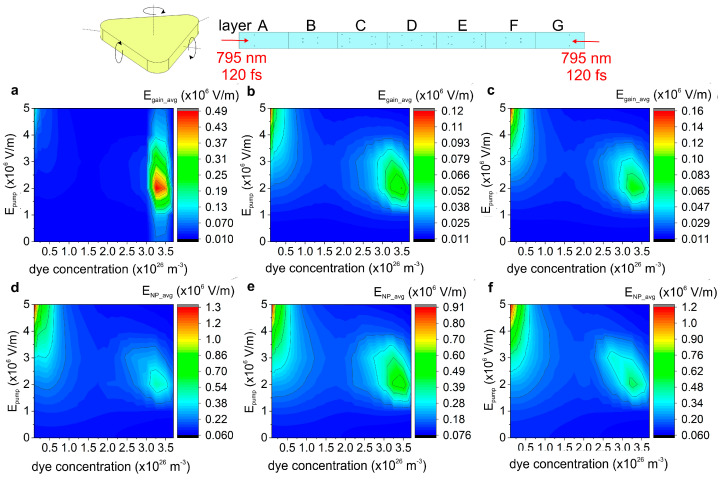
Schematic and the *E_local_* maps of the inspected nanoprism number density distributions. The average local **E**-field (**a**–**c**) inside the volume of the gain medium and (**d**–**f**) on the surface of the nanoprisms as a function of the dye molecule concentration and the pump **E**-field strength (*E_pump_*) in cases of (**a**,**d**) uniform, (**b**,**e**) Gaussian and (**c**,**f**) adjusted nanoresonator number density distribution. Inset: a schematic figure of an individual nanoprism and the vertical cross-section of seven layers in a pulse-scaled target, implanted with nanoprisms and doped with dye optionally, illuminated by two counter-propagating short-pulses.

The common target medium was a 21 µm thick urethane dimethacrylate (UDMA, Sigma Aldrich, Co., St. Louis, MO, USA) polymer slab, divided into seven, uniformly 3 µm thick, consecutive layers. The target was seeded with 70 nanoprisms made of gold in random orientation and position (inset in Figure 1), hence the density of the total target was 3.33 1/µm^3^.

The geometry of the nanoprisms was tuned to ensure resonance matching with the central wavelength of the laser pulse (Figure S4 in the Supplementary Materials). The thickness of the nanoprisms was a predefined 10 nm, while the base of the triangular antenna made of gold was tuned to 82 nm to ensure resonance at 795 nm. Additionally, the nanoprisms were coated with a thiol spacer layer according to the preparation process, which separates the dye from the gold nanoprisms. At such 1.8 nm separations, dye fluorescence quenching is strongly suppressed, while the radiative-rate enhancement associated with the plasmonic near-field remains the dominant effect. Therefore, although both phenomena coexist, the overall behavior in this geometry is expected to be governed primarily by near-field–induced local density of states enhancement.

This nanoprism geometry was selected because it can be fabricated with high reproducibility using an established experimental method, resulting in well-defined sharp tips, controlled thickness, and tunable resonance in the near-infrared [31]. The triangular nanoprisms exhibit strong enhancement stemming from the lightning-rod effect at their sharp tips, intermediate orientation-dependent plasmon resonance, and localized hot spots that are advantageous for near-field–boosted energy deposition. Their near-field enhancement is maximized, when the incident electric field is aligned with the long axis terminating in the sharpest corner. As the inset in Figure 1 shows, the random orientation means that the nanoprisms were rotated along three axes, resulting in complex hot spot distributions along the target. The achievable considerable near-field enhancement (*NFE*) does not rely exclusively on the relatively small radius of the curvature of the nanoprisms, though it plays an important role in the local field enhancement.

Three different nanoresonator distributions were examined: uniform, single-peaked Gaussian, and adjusted distribution, since our previous studies proved that they compensate for the absorption losses in uniform—Gaussian—adjusted succession [28,29]. The Gaussian distribution is defined by a fixed analytic function, namely, the nanoresonator number density changes along the propagation direction according to a Gaussian profile. The introduced adjusted nanoprism number density distribution is obtained via a constrained optimization procedure designed to enhance spatial uniformity of the near-field and power loss, as well as of the deposited energy (see Supplementary Materials). The adjusted distribution is not defined by a closed-form analytic function, but results from minimizing a composite objective function via changing the nanoprism number density and dye concentration distribution layer by layer in the proposed target, using balanced criteria in a penalty approach [32].

The targets seeded with these distributions were illuminated by two counter-propagating 120 fs short-pulses, with a central wavelength of 795 nm, in order to make the results comparable with experiments that are in progress with a Ti: Sapphire laser [33].

Besides the passive targets, their active counterparts were also considered, where the polymer was doped with a laser dye (LDS 798). In the 4-level gain model, the dye-doped polymer medium acts as an optical amplifier, whose response is incorporated through a complex, pump- and probe-dependent permittivity, *ε*(*ω*,*E_pump_*,*E_probe_*) = *ε*′(*ω*) + i*ε*″(*ω*,*E_pump_*,*E_probe_*), where the imaginary part *ε*″ becomes less positive—thereafter locally negative—by increasing pumping (Figure S5 in the Supplementary Materials). This modification inherently changes the effective refractive index, the absorption efficiency, and the local gain–loss balance throughout the complete target. 

Since the nanoprisms do not resonate at the pump wavelength, the gain modification occurs predominantly in the background medium, which then influences the plasmonic response at the probe wavelength as follows: (i) increased local pump-induced population inversion in regions of larger near-field, (ii) reduction in loss (or achievement of local net gain) that partially (over)compensates the absorption, (iii) enhanced near-field in the local environment, and (iv) opening stimulated emission channels and allowing for lasing behavior. As a result, each distribution exhibits its unique characteristic manifestation of localized random-lasing-like amplification, which governs the uniformity metrics differently.

A numerical pump-and-probe simulation was performed using a 532 nm monochromatic CW pump beam and a 795 nm CW probe beam, following the method detailed in our previous studies [19,29]. Via steady-state modeling, the average local **E**-field on the surface of the nanoprisms and in the volume of the gain medium along the target was mapped over the pump **E**-field strength (*E_pump_*) and dye concentration (*c*) parameter plane. Based on the maps taken primarily using a target with uniform nanoprism number density and dye molecule concentration distribution, a pump **E**-field strength of 2 × 10^6^ V/m and a dye concentration of 3.25 × 10^26^ m^−3^ were used in the active uniform target. With these pump and dye parameters, the local **E**-field is simultaneously enhanced both in the gain medium and on the surface of the nanoprisms (Figure 1a,d). During the next step, the number density distribution profile was modified. It was proven that the local **E**-field was efficiently enhanced at the selected pump **E**-field strength and dye concentration, when the triangular nanoresonator number density distribution was modified from uniform to either Gaussian (Figure 1b,e) or to an adjusted (Figure 1c,f) distribution. Considering the correspondence of **E**-field maxima above this parameter plane, the optimal parameters were adopted for time-dependent simulations. Importantly, the pump and probe intensity is slightly and considerably below the damage threshold of the nanoprisms (*E_damage_* = 4 × 10^6^ V/m [33]), when the pump and probe **E**-field are 2 × 10^6^ V/m and 10^4^ V/m, respectively. Although the damage-threshold values reported in Ref. [34] were measured for gold nanorods, these are considered as a reasonable approximation, since no experimentally verified threshold data exist for the same type of gold nanoprisms at wavelengths and pulse durations comparable to those used in the present study.

The saturation behavior does not prevent accessing the advantages of the gain medium, as there are several examples in the literature, where active systems pumped above the *E_saturation_* are still operable as amplifiers and plasmonically enhanced nanolasers [19]. The near-field is modified also at the pump, but the nanoprisms are not resonant at this wavelength; therefore, the local field is well below the saturation threshold on average in the target. Although the maximal local **E**-field in the gain medium might be larger than the *E_saturation_* = 1.6 × 10^6^ V/m at the boundary layers, the volume fraction of the above saturation regions was very low (*dV*/*V* = 0.70%). Accordingly, the inversion values remain below the saturation values on the average, while above saturation values are reached only in the target boundary layers at the pump wavelength.

In the case of the adjusted nanoprism number density distribution, the dye molecule concentration distribution was also modified with the criterion that the average concentration of the dye remains the same as in the case of uniformly doped targets.

The time evolution of the power loss (*PL*(*t*)) and average near-field enhancement (*NFE*(*t*)) was determined in each layer. For the complete study of the deposited energy (*E(t)*), please see the Supplementary Materials. The time evolution of the standard deviation was inspected to determine the value (*δ*_min_PL/E/NFE_), time-instant (*t_min_PL/E/NFE_*), and delay compared to the time-instant (*t_overlap_* = 240 fs) of the theoretical overlap of counter-propagating laser pulses (Δ*t_min_PL/E/NFE_* = |*t_overlap_* − *t_min_PL/E/NFE_*|) of the minimal standard deviation of the inspected quantities. 

The power loss (and also the deposited energy) was integrated until *t_overlap_* specifying the values of absorbed power loss, i.e., the energy in the units of Joules (and deposited energy in the units of J∙s).

Based on the time evolution of the integrated power loss (as well as of the deposited energy, see the Supplementary Materials) and *NFE*, the average values of these quantities were calculated at *t_overlap_*, and the normalized standard deviation (*δ_PL_*, *δ_E_*, *δ_NFE_*) along the target was determined as follows:

*δ* = *standard deviation*/*average value*. (1)

The figure of merit (*FOM*) was defined as the ratio of the average value of the inspected quantity and the normalized standard deviation (*FOM_PL_* = *PL*/*δ_PL_*, *FOM_E_* = *E*/*δ_E_*, *FOM_NFE_* = *NFE*/*δ_NFE_*) (Supplementary Table S1, with corresponding numerical error based on convergence study of Figure S3).

It is important to note that the present study does not aim to model a full ICF scenario. Instead, our objective is to investigate the moderate fluence and early-time electromagnetic energy deposition induced by two counter-propagating 120 fs femtosecond pulses in micrometer-scaled targets, and to evaluate how optimized nanoprism distributions can improve the spatial uniformity of this deposition. Inspection of phenomena at higher fluences or longer timescales would require a coupled Maxwell–hydrodynamic and thermal conduction model and long simulation time, which is beyond the scope of this work due to the computational demand in the case of supercells with random nanotriangle distributions, but represents a potential direction for future studies.

## 3. Results

### 3.1. Passive Targets

#### 3.1.1. Dynamics of Power Absorption and Near-Field Enhancement

**Figure 2:**
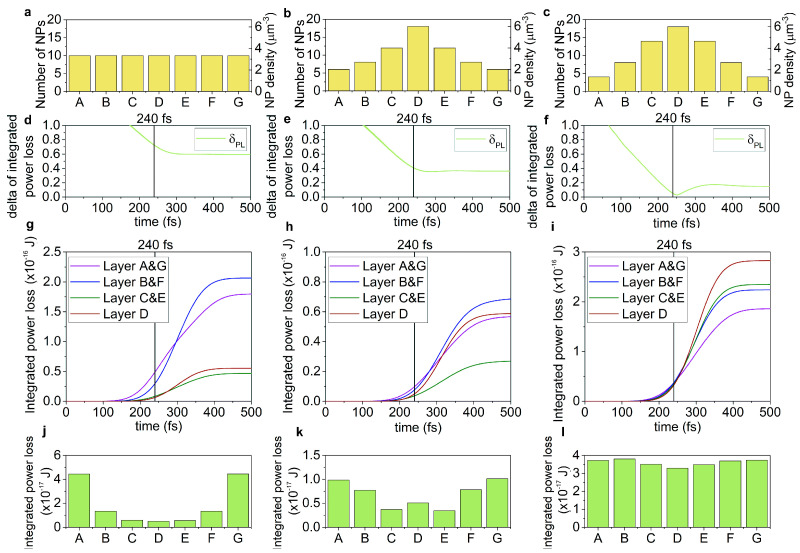
Time-dependent power loss in passive targets. The (**a**–**c**) nanoprism number density distribution along the target, the time-evolution of the (**d**–**f**) standard deviation of the power loss, and the (**g**–**i**) integrated power loss. (**j**–**l**) The distribution of the power loss integrated until 240 fs in different layers. (**a**,**d**,**g**,**j**) Uniform, (**b**,**e**,**h**,**k**) Gaussian, and (**c**,**f**,**i**,**l**) adjusted nanoprism number density distributions. (A–G indicates the seven segments of the target of uniform 3 μm thickness).

**Figure 3:**
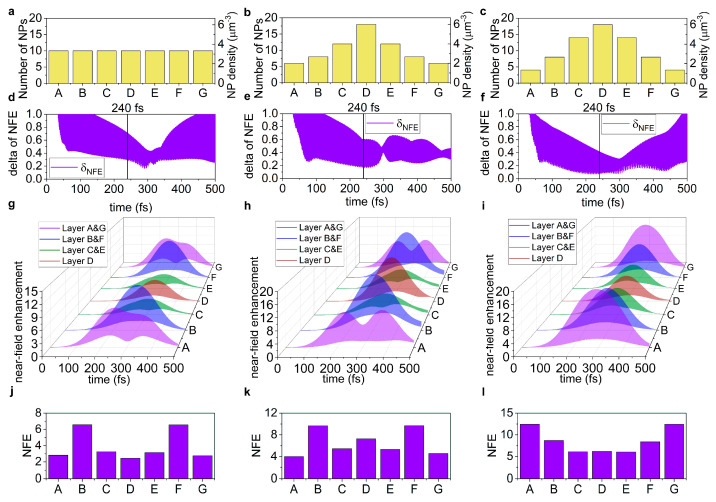
Time-dependent near-field enhancement in passive targets. The (**a**–**c**) triangular nanoresonator number density distributions along the target. The time-evolution of the (**d**–**f**) standard deviation of the instantaneous local electric field enhancement compared to the exciting electric field (*NFE*) and the (**g**–**i**) instantaneous *NFE*. (**j**–**l**) The distribution of the *NFE* in different layers at 240 fs. (**a**,**d**,**g**,**j**) Uniform, (**b**,**e**,**h**,**k**) Gaussian, and (**c**,**f**,**i**,**l**) adjusted nanoprism number density distributions. (A–G indicates the seven segments of the target of uniform 3 μm thickness).

In the passive targets, the time evolution of the power loss and near-field enhancement (*NFE*) in each distribution type is distinctly different (Supplementary Table S1, Figure 2 and Figure 3).

The uniform nanoprism number density distribution shows the largest minimal standard deviation in the power loss with the largest delay, and allows for advantageous intermediate minimal standard deviation in the *NFE*, however, also with the largest delay (Figure 2a,d,g and Figure 3a,d,g). The Gaussian nanoprism number density distribution is advantageous due to the intermediate minimal standard deviation with intermediate delay in the power loss, but has the largest minimal standard deviation in the *NFE*, though with the smallest delay (Figure 2b,e,h and Figure 3b,e,h). The advantage of the adjusted nanoprism number density distribution is that the smallest minimal standard deviation is achieved both in the power loss and in the *NFE*, with the smallest and with compromised intermediate delay, respectively (Figure 2c,f,i and Figure 3c,f,i).

#### 3.1.2. Evaluation at the Time-Instant of the Pulses’ Overlap

Intermediate averaged integrated power loss is achieved with the uniform nanoresonator number density distribution, but the standard deviation is the largest at the time-instant of counter-propagating pulses’ overlap (Supplementary Table S1, Figure 2g,j).

In the average *NFE*, the target with uniform nanoprism number density distribution is weak, as it allows for the smallest average value (3.94-fold enhancement) and the largest standard deviation (Supplementary Table S1, Figure 3g,j).

In the case of the Gaussian nanoprism number density distribution, the smallest power loss with an intermediate standard deviation can be achieved, while the *NFE* (6.55-fold) and its standard deviation are also intermediate (Supplementary Table S1, Figure 2h,k and Figure 3h,k).

The adjusted nanoprism number density distribution produces the largest power loss and *NFE* (8.61-fold), with the smallest standard deviation in both quantities (Supplementary Table S1, Figure 2i,l and Figure 3i,l).

Based on the figure of merit (*FOM_PL_*) of the power loss, the uniform nanoprism number density distribution (3.58 × 10^−17^ J) is intermediate; the least advantageous is the single-peaked Gaussian distribution (1.68 × 10^−17^ J), while the adjusted distribution (7.14 × 10^−16^ J) is the most advantageous. However, comparing the *FOM* of the *NFE* (*FOM_NFE_*), the ranking of the targets slightly modifies. The uniform nanoprism number density distribution (8.54) becomes the weakest, the Gaussian distribution (18.29) is intermediate, while the adjusted distribution (26.08) remains the most advantageous, also in the *FOM_NFE_* (Supplementary Table S1). The uniform nanoprism number density distribution is intermediate in the power loss and in the *FOM* of the power loss (*FOM_PL_*), while it is the weakest in the average *NFE*, in the standard deviation of the power loss (*δ_PL_*) and *NFE* (*δ_NFE_*), as well as in the *FOM* of the *NFE* (*FOM_NFE_*). The Gaussian nanoprism number density distribution is intermediate in the *δ_PL_* and *δ_NFE_* at 240 fs, in the average *NFE*, and in the *FOM_NFE_*, while it is the weakest in the achieved average power loss and in the *FOM_PL_*. The adjusted nanoprism number density distribution achieves the smallest standard deviation, the largest average value, and also the largest *FOM_PL_* and *FOM_NFE_*. These results prove the advantage of the adjusted nanoprism distributions in passive targets.

#### 3.1.3. Ranking of the Passive Targets

Each *FOM* is evaluated self-consistently within its own metric, and the ranking of the nanoprism distributions is performed exclusively on the basis of relative performance considering the same physical quantity category.

Consequently, the dimensional difference does not affect the comparisons or the ranking concluded based on FOMs. If every inspected quantity is equally considered in the ranking, then the nanoprism number density distributions are not unambiguously comparable. The weakest/compromised intermediate/the most preferable distribution is the uniform/Gaussian/adjusted, as it shows 7–3–0/3–6–1/0–1–9 quantities in which the specific system is the weakest, intermediate, and the most preferable. Based on the *FOM_PL_*, the distribution ranking shows Gaussian/uniform/adjusted order, while considering the *FOM_NFE_*, the uniform/Gaussian/adjusted order appears, proving that the most advantageous is the adjusted distribution, in accordance with the intuitive expectations (Supplementary Tables S2 and S3). The non-uniform nanoprism number density distributions possess better characteristics already in passive targets.

### 3.2. Active Targets

#### 3.2.1. Dynamics of the Power Absorption and the Near-Field Enhancement

**Figure 4:**
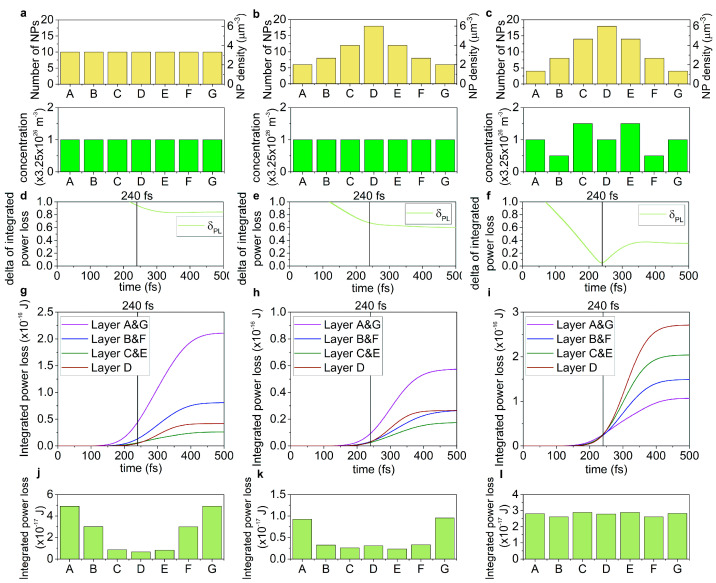
Time-dependent power loss in active targets. The (**a**–**c**) nanoprism number density and dye molecule concentration distribution along the target. The time-evolution of the (**d**–**f**) standard deviation of the power loss and the (**g**–**i**) integrated power loss. (**j**–**l**) The distribution of the power loss integrated until 240 fs in different layers. (**a**,**d**,**g**,**j**) Uniform, (**b**,**e**,**h**,**k**) Gaussian, and (**c**,**f**,**i**,**l**) adjusted nanoprism number density distributions. The dye concentration is adjusted only for the adjusted nanoprism number density distribution in (**c**,**f**,**i**,**l**). (A–G indicates the seven segments of the target of uniform 3 μm thickness).

**Figure 5:**
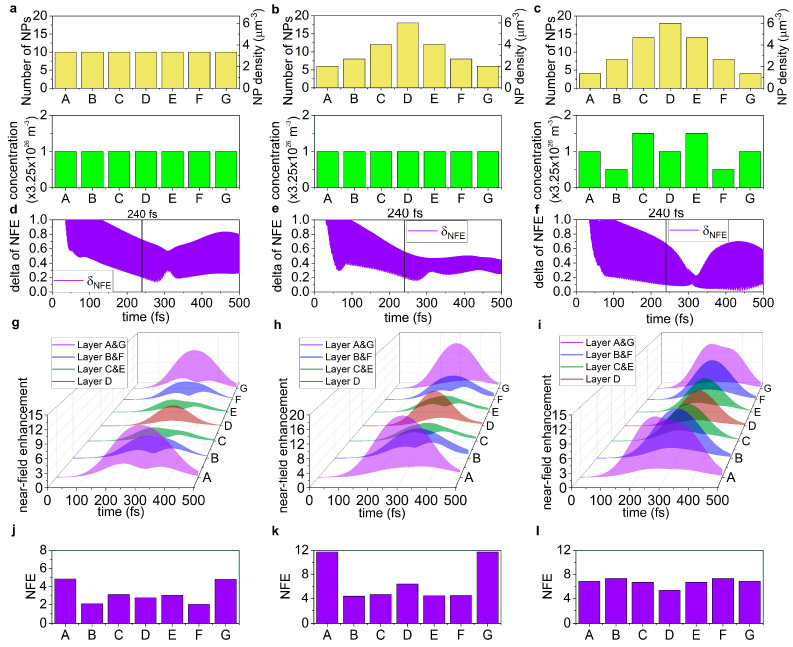
The time-dependent near-field enhancement in active targets. The (**a**–**c**) nanoprism number density and dye molecule concentration distributions along the target. The time-evolution of the (**d**–**f**) standard deviation of the instantaneous local electric field enhancement compared to the exciting electric field (*NFE*) and the (**g**–**i**) instantaneous *NFE*. (**j**–**l**) The distribution of the *NFE* at 240 fs in different layers. (**a**,**d**,**g**,**j**) uniform, (**b**,**e**,**h**,**k**) Gaussian, and (**c**,**f**,**i**,**l**) adjusted nanoprism number density and dye concentration distributions. (A–G indicates the seven segments of the target of uniform 3 μm thickness).

In the active targets, the time evolution of the power loss and *NFE* in each target type is distinctly different, as in the case of the passive targets (Supplementary Table S1, Figure 4 and Figure 5). The uniform nanoprism number density distribution in the active target shows the largest minimal standard deviation in the power loss with intermediate delay, and intermediate minimal standard deviation in the *NFE* with intermediate delay (Figure 4a,d,g and Figure 5a,d,g). Compared to the uniform nanoprism number density distribution in a passive target, the minimal standard deviation is considerably increased in the power loss, while it is slightly decreased in the *NFE*. The delay of minimal standard deviation decreased both in the power loss and in the *NFE*, compared to the uniform nanoprism number density distribution in the passive target (Supplementary Table S1).

In the case of the single-peaked Gaussian nanoprism number density distribution in the active target, the minimal standard deviation of the power loss is intermediate, but with the largest delay, while the minimal standard deviation of the *NFE* is the largest, though it is taken on advantageously with the smallest delay (Figure 4b,e,h and Figure 5b,e,h). Compared to the Gaussian nanoprism number density distribution in the passive target, the minimal standard deviation is considerably increased in the power loss, and it is slightly decreased in the *NFE*, while the delay is considerably/slightly increased for the minimal standard deviation in the power loss/*NFE* (Supplementary Table S1).

The advantage of the adjusted nanoprism number density and dye concentration distribution in the active target is that the smallest minimal standard deviation in the power loss is achieved with the smallest delay; moreover, the smallest standard deviation is reached in the *NFE*, though with the largest delay among the active targets. These results indicate that the temporal characteristics are predominantly improved by adjusting the nanoprism number density and dye concentration distribution.

Compared to the adjusted nanoprism number density distribution in the passive target, the minimal standard deviation is slightly increased in the power loss, and it is significantly decreased in the *NFE*. Although only the latter is improved, both are the smallest among the minimal deviations taken in active targets. The delay of the minimal standard deviation is decreased in the power loss compared to the adjusted distribution in the passive target, while it is significantly increased in the *NFE*. The former indicates that dye doping might be advantageous in terms of delay as well (Supplementary Table S1).

Dye doping of targets embedding uniform and adjusted nanoprism number density distributions is advantageous in decreasing the delay between the time instant of minimal standard deviation and the theoretical overlap of counter-propagating pulses in the case of power loss and *NFE* (as well as of the deposited energy, see Supplementary Materials), except for the delay of the minimal standard deviation in the *NFE* in the adjusted distribution, which is increased compared to its counterpart registered in the case of a passive target. The single-peaked Gaussian nanoprism number density distribution is remarkably different, as the dye doping results in increased delay of the minimal standard deviation of all quantities.

Dye doping is also advantageous in all inspected nanoprism number density distributions, considering the decreased minimal standard deviation of *NFE*; however, improvement of the uniformity of the power loss that can be achieved throughout the pulses’ overlap in active targets is the subject of further study (Supplementary Tables S1 and S2).

#### 3.2.2. Evaluation at the Time-Instant of Pulse-Overlap

Similarly to the uniform nanoprism number density distribution in the passive target, intermediate average integrated power loss is achieved in the active medium, though with the largest standard deviation, while the average *NFE* (3.23-fold) is the smallest, with intermediate standard deviation at the theoretical overlap of the counter-propagating pulses (Supplementary Table S1, Figure 4g,j and Figure 5g,j). 

Compared to the uniform nanoprism number density distribution in the passive target, both the power loss and the *NFE* are slightly decreased, the standard deviation of the power loss (*δ_PL_*) is increased, while for the *NFE* (*δ_NFE_*) it is decreased (Supplementary Table S1). The smallest power loss is observed in the case of the Gaussian nanoprism number density distribution, with intermediate standard deviation, while the largest *NFE* (6.83-fold) can be achieved; however, it is compromised with the largest standard deviation (Supplementary Table S1, Figure 4h,k and Figure 5h,k). Compared to the Gaussian nanoprism number density distribution in a passive target, the power loss is slightly decreased, though the *NFE* is slightly increased; however, the standard deviation of both quantities is increased (Supplementary Table S1). The adjusted nanoprism number density and dye concentration distribution allowed for the largest power loss with the smallest standard deviation, though it exhibited intermediate *NFE* (6.74-fold), similarly with the smallest deviation (Supplementary Table S1, Figure 4i,l and Figure 5i,l). Compared to the adjusted distribution in the passive target, the power loss and *NFE* are slightly decreased, and the standard deviation of the power loss (*δ_PL_*)/*NFE* (*δ_NFE_*) is slightly/significantly decreased, which indicates that the dye doping has well-defined advantages in both quantities in achieving a larger degree of uniformity along the target (Supplementary Table S1).

In the case of uniform and Gaussian nanoprism number density distributions, the integrated power loss decreases, while the standard deviation increases compared to their counterparts in the passive target.

In the target with adjusted nanoprism number density and dye concentration distribution, the power loss decreases, but its standard deviation also decreases; the latter indicates that the dye seeding can be advantageous in achieving uniformity in the power loss along the target (Supplementary Tables S1 and S3).

Compared to their passive counterparts, the *NFE* and its standard deviation (*δ_NFE_*) are decreased in the uniform and adjusted distributions, while in the Gaussian distribution, both quantities are increased (Supplementary Tables S1 and S3). These results indicate nanoprism number density distribution dependent, and compromised advantages of the dye doping, which manifest themselves in complementary improvement either in the standard deviation or in the value of the *NFE*. Based on the *FOM* of the power loss (*FOM_PL_*), the uniform nanoprism number density distribution is intermediate (1.99 × 10^−17^ J), the Gaussian distribution is the weakest (7.02 × 10^−18^ J), while the adjusted nanoprism number density and dye concentration distribution is the most advantageous (5.76 × 10^−16^ J). This ranking slightly modifies when the *FOM_NFE_* is compared, similarly to the passive targets. Namely, the *FOM_NFE_* in the uniform distribution becomes the least advantageous (8.97), in the Gaussian distribution is intermediate (13.63), while in the adjusted nanoprism number density and dye concentration distribution remains the most beneficial (72.35) (Supplementary Table S1).

Compared to their counterpart distributions in the passive target, every *FOM* is decreased, except the *FOM_NFE_* in the uniform and adjusted distributions, which indicates nanoprism number density distribution specific advantages of dye seeding. In the active targets, the ranking of the different inspected distributions remains the same in both *FOM*s, as in the passive targets (Supplementary Tables S1 and S3). At 240 fs, the uniform number density distribution is intermediate in the power loss, *FOM_PL,_* and the standard deviation of the *NFE* (*δ_NFE_*), while it is the weakest in the standard deviation of the power loss (*δ_PL_*), mean *NFE,* and the *FOM_NFE_*. By the Gaussian number density distribution the largest *NFE* is achieved among the active targets, while it is intermediate in the standard deviation of the power loss (*δ_PL_*) and *FOM_NFE_*. However, the Gaussian number density distribution shows the smallest power loss, *FOM_PL_*, and the largest standard deviation in the *NFE* (*δ_NFE_*). The adjusted nanoprism number density and dye concentration distribution is the most advantageous due to the largest power loss, smallest standard deviations in the power loss and *NFE,* and the largest *FOM*s. It is intermediate only in the average *NFE*. Considering the power loss, the adjusted nanoprism number density and dye concentration distribution in the active target show the sole advantage of possessing a standard deviation smaller than its counterpart in the passive target. Considering the *NFE*, dye doping is advantageous in increasing the average *NFE* in the Gaussian number density distribution, and it is also beneficial in achieving a smaller standard deviation and larger *FOM_NFE_* in the uniform and adjusted distributions compared to their counterparts in passive targets. On the timescale of the short-pulse overlap, coupling effects are already at play, as the average distance of the nanoprism is 1.2 μm, which is 40 times smaller compared to the light propagation distance during 240 fs in UDMA. Although the coupling on the specific random nanoprism distribution is not of primary importance, the quantity selective advantage of the gain medium might be explained by the intermediate orientation selective excitation and coupling efficiency of nanoprisms.

#### 3.2.3. Ranking of the Active Targets

Similarly to the passive targets, the ranking is not balanced. When every inspected quantity is equally considered, the only difference is that the Gaussian/adjusted (implying dye concentration) number density distribution becomes significantly/slightly less advantageous than the uniform/Gaussian distribution. 

Namely, the weakest/compromised intermediate/the most preferable is the uniform/Gaussian/adjusted (implying dye concentration) number density distribution, as it shows 4–6–0/5–3–2/1–1–8 quantities; in which the specific system is the weakest—intermediate—the most preferable. Based on the *FOM_PL_*, the nanoprism distribution ranking shows Gaussian/uniform/adjusted order, while considering the *FOM_NFE_* uniform/Gaussian/adjusted order appears, so the most advantageous is the adjusted number density distribution, in accordance with the intuitive expectations, analogously with passive targets, but with the additionally adjusted dye concentration distribution (Supplementary Tables S2 and S3). The non-uniform nanoprism number density distributions possess better characteristics also in active targets.

## 4. Conclusions

A comparative study was conducted on different nanoprism number density distributions embedded into passive and active targets. A specific parameter region was identified by sweeping the dye molecule concentration and pump **E**-field strength, where a large average local **E**-field can be achieved both in the gain medium and on the nanoprisms’ surfaces, in each inspected distribution.

In the case of passive targets, using a uniform nanoprism number density distribution is the least efficient method to ensure uniform power loss and near-field along the target based on the analyzed quantities. However, single-peaked Gaussian nanoprism number density distribution proved to be advantageous due to that the smallest delay of the minimal standard deviation in the *NFE* can be achieved. The Gaussian distribution is also advantageous in the amount of deposited energy. The adjusted nanoprism number density distribution is the most advantageous, due to the smallest minimal standard deviation in all inspected quantities, the smallest delay of the minimal standard deviation in the power loss (and deposited energy, see Supplementary Material) the largest integrated power loss and reached *NFE* value, and the smallest standard deviation at 240 fs in the power loss, *NFE* (and deposited energy see Supplementary Material). Furthermore, the largest *FOMs* can be achieved in every inspected quantity with the adjusted distribution in the case of passive targets. Based on these results, the adjusted nanoprism number density distribution is proposed among the passive targets (Supplementary Tables S1–S3).

Similarly, in the active targets, the uniform nanoprism number density distribution is the least advantageous, though it becomes intermediate in more and remains the weakest in fewer quantities, compared to the counterpart distribution in the passive target.

The Gaussian nanoprism number density distribution has several advantages, namely the smallest delay of the minimal standard deviation in the *NFE*, and the largest *NFE* (and deposited energy, see Supplementary Material) are achieved by using a single-peaked Gaussian distribution. However, on average, the adjusted nanoprism number density distribution is the most advantageous, similarly to the passive targets, with the additionally adjusted dye concentration distribution. This is due to that the adjusted distributions allowed for the smallest minimal standard deviation in all inspected quantities, the smallest delay of the minimal standard deviation in the power loss (and deposited energy, see Supplementary Material), the largest integrated power loss, the smallest standard deviation at 240 fs in all inspected quantities, and also the largest *FOM*s. Based on these results, the adjusted nanoprism number density and dye concentration distributions are proposed in the active targets (Supplementary Tables S1–S3, about energy-related data, please see Supplementary Materials).

The modification of the dielectric properties at the probe wavelength proves that the composite acts as an amplifier. Accordingly, the dye-doped complex target strongly enhances the local field and results in power absorption modification. 

All these phenomena depend on time, and the net impact was described based on the presented time-dependent results. These time-dependent results prove that doping with the dye of the target embedding the nanoprism distribution is not uniformly advantageous compared to the passive counterparts. In the case of the uniform and adjusted nanoprism number density distributions doped with uniformly distributed dye, the minimal standard deviation of the *NFE*, and the delay of the minimal standard deviation in the power loss and deposited energy become smaller; moreover, the standard deviation of the *NFE* at 240 fs is also smaller, while the *FOM_NFE_* is larger. In addition to this, in the uniform nanoprism number density and dye concentration distributions, the delay of the minimal standard deviation in the *NFE*, while in the adjusted nanoprism number density and dye concentration distributions, the standard deviation of the power loss and deposited energy at 240 fs becomes smaller compared to their passive counterparts.

In the case of the Gaussian nanoprism number density distribution, using uniformly distributed dye is already advantageous in facilitating a smaller minimal standard deviation of the *NFE*, similar to the other two distributions, as well as in allowing for a larger mean *NFE* value at 240 fs exclusively (Supplementary Tables S1–S3). 

The advantage of dye doping is more strongly dependent on both nanoprism number density distribution and the inspected quantity than in the previously studied nanoshell seeded targets [28,29], due to the intermediate orientation-selective excitation and coupling efficiency characteristic of triangular nanoresonators.

Comparing every inspected target type and distributions, the passive target with an adjusted number density distribution is proposed, when the target is seeded with asymmetric nanoprisms, which is closely followed by its active counterpart in the global ranking. The standard deviation of the power loss (*δ_NFE_*) and energy (*δ_E_*) (*NFE* (*δ_NFE_*)) at 240 fs is reduced as well, and the *FOM_NFE_* (and *FOM_E_*, see Supplementary Material) are increased in the uniform—single-peaked Gaussian—adjusted number density distribution order, as is expected in both (passive) targets. The other characteristic values—including the minimal standard deviation of *NFE* (standard deviation of the *NFE* at 240 fs) as well as the *FOM_PL_* in (active) both targets—exhibit the single-peaked Gaussian—uniform—adjusted number density distribution order. This can be explained by the fact that the uniform and Gaussian distributions have more predefined constraints and thus only a compromised uniformity can be achieved. In the case of the adjusted distribution, the nanoprism number density and dye concentration distributions were simultaneously adjusted to minimize the standard deviations measured at 240 fs, and thus to make the integrated power loss and *NFE* as uniform and as high as possible at the theoretical time of counter-propagating pulses overlap.

In the passive targets, the improved power loss and *NFE* uniformity imply an increase both in integrated power loss and in *NFE*, while in the active target, only the power loss uniformity improvement is accompanied by increased integrated power loss. This can be explained by the fact that all active targets are compromised, with balanced advantages and disadvantages. By doping the targets with dye, the standard deviation of the *NFE* (power loss (and deposited energy, see Supplementary Material)) at 240 fs was reduced except for the Gaussian nanoprism number density distribution (in the adjusted nanoprism number density and dye concentration distribution), but the power loss, deposited energy, and achieved *NFE* were smaller than in the counterpart (except for the Gaussian) distributions in the passive target.

According to the composite objective function in non-uniform distributions, the *FOM* was improved for all quantities compared to the uniform distribution in passive and active targets (except the *FOM_PL_* in Gaussian nanoprism number density distributions). 

The adjusted distributions in active targets outperform the uniform number density distribution in passive targets in all quantities, except for the delay of the minimal standard deviation in the *NFE*. Moreover, the adjusted distributions in the active target outperform even the counterpart passive target in the minimal standard deviation of the *NFE*, in the delay of the minimal standard deviation in the power loss and deposited energy, and in the standard deviation of all inspected quantities at 240 fs. Slight/significant *FOM_NFE_* improvement is achieved via dye doping for the uniform/adjusted distribution types compared to their counterpart passive targets. Joint optimization with composite objective functions and adding more constraints is a subject of further studies to precisely tune both the nanoresonator number density and dye concentration distributions in order to achieve specific criteria of applications.

## Data Availability

The original contributions presented in this study are included in the article/Supplementary Material. Further inquiries can be directed to the corresponding author.

## Supplementary Materials

The following supporting information can be downloaded at: https://www.mdpi.com/article/10.3390/nano15231801/s1. Figure S1: Time-dependent deposited energy in passive targets; Figure S2: Time-dependent deposited energy in active targets.; Figure S3: Mesh convergence study in a typical configuration.; Figure S4: Absorption, scattering, and extinction cross-section of the used nanotriangles. Figure S5: Spectra of the optical properties of the dye medium, absorptions, and near-field enhancement.; Table S1: Targets seeded with gold nanoprisms.; Table S2: The ranking of the inspected targets in the minimal standard deviation and in its delay. Table S3: The ranking of the inspected targets in the average value, along with the target and its standard deviation.

## Abbreviations

The following abbreviations are used in this manuscript:

NFE	Near-field enhancement
FOM	Figure of merit
NP	Nanoparticle
LSPR	Localized surface plasmon resonance
QD	Quantum dot
FWHM	Full-width-at-half-maximum
PL	Power loss
CW	Continuous wave
SLR	Surface lattice resonance
UDMA	Urethane dimethacrylate
