# Supplementary material for: Plasmonic Nanoprism Distributions to Promote Enhanced and Uniform Energy Deposition in Passive and Active Targets
Source: Nanomaterials (Basel). 2025 Nov 29;15(23):1801. doi: 10.3390/nano15231801 (PMC12692952; doi:10.3390/nano15231801)
Supplement: Supplementary file 1 [file nanomaterials-15-01801-s001.zip › nanomaterials-3981521-supplementary.pdf]

Supplementary Materials

Plasmonic nanoprism distributions to promote enhanced and uniform energy deposition in passive and active targets

Dávid Vass[1,2], Emese Tóth[1,2], András Szenes[1,2], Balázs Bánhelyi[2,3], István Papp[2,4], Tamás Sándor Biró[2], László Pál Csernai[2,4,5], Norbert Kroó[2], and Mária Csete[1,2,*]

[1] *Department of Optics and Quantum Electronics, University of Szeged, 6720 Szeged, Hungary*
[2] *Wigner Research Centre for Physics, 1121 Budapest, Hungary*
[3] *Department of Computational Optimization, University of Szeged, 6720 Szeged, Hungary*
[4] *Department of Physics and Technology, University of Bergen, 5007 Bergen, Norway*
[5] *Frankfurt Institute for Advanced Studies, 60438 Frankfurt am Main, Germany*

Energy Deposition

Passive Targets

Dynamics of Deposited Energy

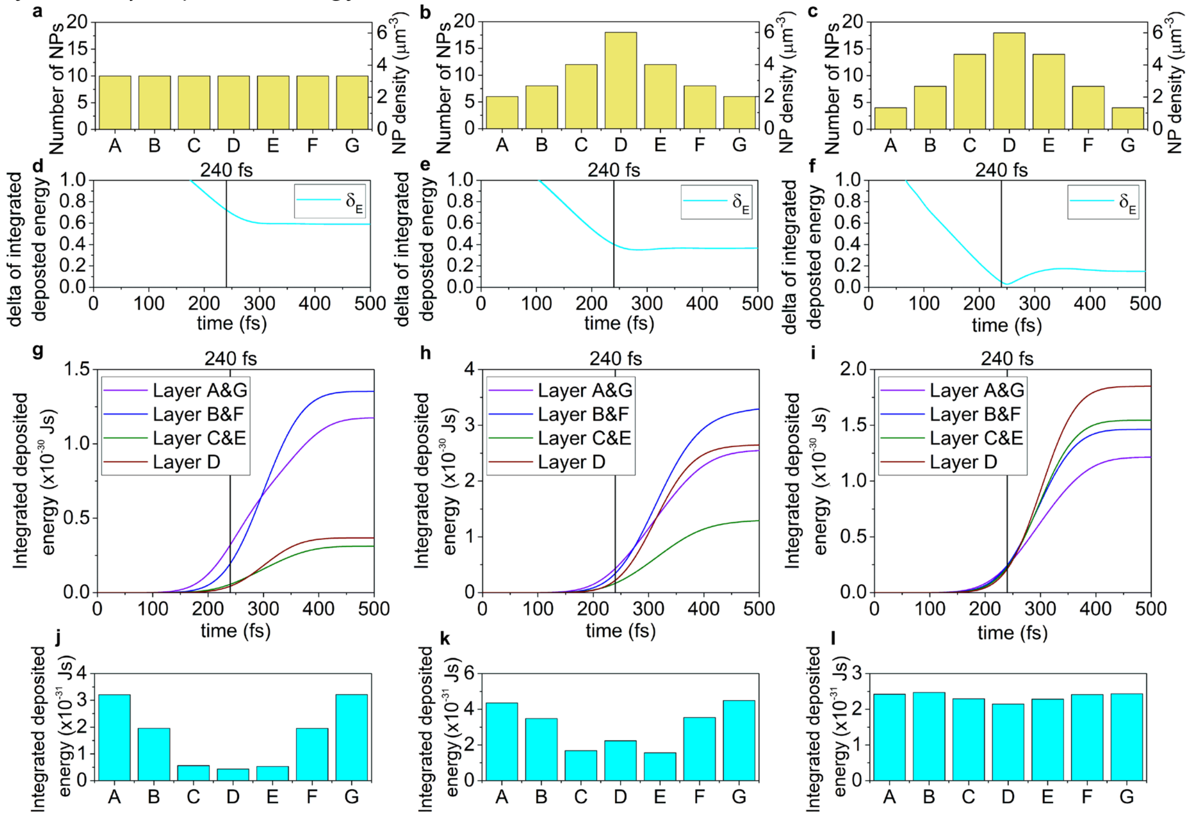

Figure S1. Time-dependent deposited energy in passive targets. The (**a-c**) nanoprism number density distribution along the target. The time-evolution of the (**d-f**) standard deviation of the deposited energy and the (**g-i**) integrated deposited energy. (**j-l**) The distribution of the deposited energy in different layers integrated until 240 fs. (**a,d,g,j**) Uniform, (**b,e,h,k**) Gaussian and (**c,f,i,l**) adjusted nanoprism number density distributions. (A-G indicates the seven segments of the target of uniform 3 μm thickness)

The uniform nanoprism number density distribution shows the largest minimal standard deviation in the deposited energy with the largest delay (Supplementary Table S1, Figure S1a,d,g). The Gaussian nanoprism number density distribution is better due to the intermediate minimal standard deviation taken with intermediate delay in the deposited energy (Supplementary Table S1, Figure S1b,e,h).

The advantage of the adjusted nanoprism number density distribution is the smallest minimal standard deviation in the deposited energy, taken with the smallest delay (Supplementary Table S1, Figure S1c,f,i).

Evaluation at the Time-Instant of Pulses' Overlap

The integrated deposited energy is the smallest in uniform nanoprism number density distribution, moreover the standard deviation at 240 fs is the largest (Supplementary Table S1, Figure S1g,j). In case of the Gaussian nanoprism number density distribution the deposited energy is the largest and the standard deviation is intermediate (Supplementary Table S1, Fig S1h,k). The adjusted nanoprism number density distribution shows intermediate deposited energy, and the smallest standard deviation (Supplementary Table S1, Figure S1i,l)

Based on the *FOM* of the deposited energy, the uniform distribution (2.35×10^{-31} Js) is the weakest, the Gaussian distribution is intermediate (7.60×10^{-31} Js), while the adjusted distribution (4.79×10^{-30} Js) is the most advantageous (Supplementary Table S1).

In summary, the uniform nanoprism number density distribution is the weakest in the average value, standard deviation and the *FOM* of the deposited energy. The Gaussian nanoprism number density distribution is intermediate in the standard deviation and in the *FOM*, and the most advantageous in the average deposited energy. The adjusted nanoprism number density distribution is the most advantageous on the average, due to its smallest standard deviation and largest *FOM*, though the average value is intermediate.

Ranking of the Passive Targets

If every inspected quantity is equally considered in the ranking, then the distributions are not comparable. The weakest/compromised intermediate/the most preferable is the uniform/Gaussian/adjusted nanoprism number density distribution, as it shows 5–0–0/0–4–1/0–1–4 quantities; in which the specific system is the weakest—intermediate—the most preferable. Based on the *FOM* of the deposited energy, the distribution ranking shows uniform/Gaussian/adjusted order, so the most advantageous is the adjusted nanoprism number density distribution, in accordance with the intuitive expectations.

Active Targets

Dynamics of Deposited Energy

The uniform nanoprism number density distribution in active target shows the largest minimal standard deviation in the deposited energy with intermediate delay (Supplementary Table S1, Figure S2a,d,g). Compared to the uniform distribution in passive target, the minimal standard deviation is increased, while the delay is decreased. The Gaussian nanoprism number density distribution in active target is advantageous due to the intermediate minimal standard deviation in the deposited energy, but it has the largest delay (Supplementary Table S1, Figure S1b,e,h). Compared to the Gaussian distribution in passive target, both the minimal standard deviation and its delay is increased. The advantage of the adjusted nanoprism number density and dye concentration distribution is that the smallest minimal standard deviation in the deposited energy is achieved with the smallest delay in the active targets, as a result the temporal characteristics are the most advantageous (Supplementary Table S1, Figure S2c,f,i). Compared to the adjusted nanoprism number density distribution in the passive target, the minimal standard deviation is increased in the deposited energy, while the delay of it is decreased.

Evaluation at the Time-Instant of Pulses' Overlap

The integrated deposited energy is the smallest in uniform nanoresonator number density distribution, moreover the standard deviation is the largest (Supplementary Table S1, Figure S2g,j). In case of the Gaussian nanoprism number density distribution the deposited energy takes on the largest value, while its standard deviation is intermediate (Supplementary Table S1, Figure S2h,k). The adjusted nanoprism number density and dye concentration distribution resulted in intermediate deposited energy and the smallest standard deviation (Supplementary Table S1, Figure S2i,l). In case of uniform and Gaussian nanoprism number density distributions in active target, the deposited energy is smaller, while the standard deviation is larger compared to their counterparts in passive target.

In the adjusted nanoprism number density and dye concentration distribution though the deposited energy is decreased, its standard deviation is also smaller, which indicates that the dye doping is advantageous in achieving more uniform deposited energy distribution along the target.

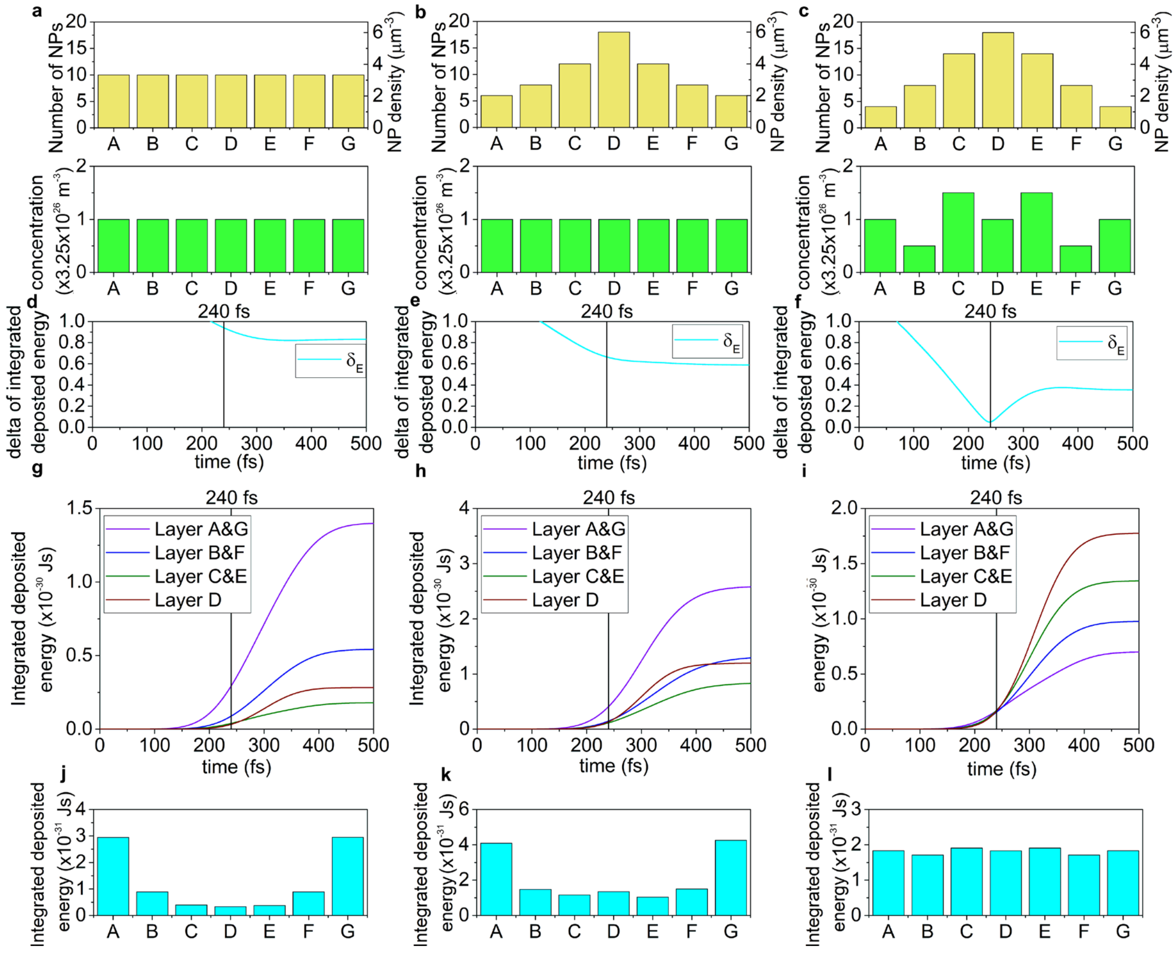

Figure S2. The time-dependent deposited energy in active targets. The (**a-c**) nanoprism number density and dye molecule concentration distribution along the target. The time-evolution of the (**d-f**) standard deviation of the deposited energy and the (**g-**i) integrated deposited energy. (**j-l**) The distribution of the deposited energy in different layers integrated until 240 fs. (**a,d,g,j**) Uniform, (**b,e,h,k**) Gaussian and (**c,f,i,l**) adjusted nanoprism number density distributions. The dye concentration is adjusted only for adjusted nanoprism number density distribution in (**c,f,i,l**). (A-G indicates the seven segments of the target of uniform 3 μm thickness)

Based on the *FOM* of the deposited energy the uniform nanoprism number density distribution is the weakest (1.33×10^{-31} Js), the Gaussian distribution is intermediate (3.20×10^{-31} Js), and the adjusted nanoprism number density and dye concentration distribution is the most advantageous (3.78×10^{-30} Js) (Supplementary Table S1). The *FOM* of the energy is smaller in every inspected distribution compared to their counterparts in passive target. Similarly to the passive targets, the adjusted nanoprism number density distribution is the most advantageous also in active targets.

The uniform nanoresonator number density distribution is the least advantageous in the active target because of the smallest deposited energy, *FOM* of the deposited energy, and the largest standard deviations of the deposited energy.
The Gaussian nanoprism number density distribution in the active target allows for the largest deposited energy, while it is intermediate in its standard deviation and *FOM*.
The unambiguous advantage of the adjusted nanoprism number density and dye concentration distribution in the active target is indicated by the largest *FOM* and the smallest standard deviation of the deposited energy, though it is intermediate in the average deposited energy.
Based on the deposited energy, the inspected nanoprism number density distributions in passive targets outperform their counterparts in active targets in the average value, the standard deviation and also in the *FOM*, except the adjusted nanoprism number density and dye concentration distribution in active target, which allows for standard deviation smaller than that achievable via counterpart distribution in passive target.

Ranking of the Active Targets
Similarly to the passive targets, the ranking is not balanced, when every inspected quantity is equally considered, the only difference is that the adjusted nanoprism number density and dye concentration distribution becomes more advantageous, than the Gaussian distribution. Namely, the weakest/compromised intermediate/the most advantageous is the uniform/Gaussian/adjusted (implying dye concentration) number density distribution, as it shows 4–1–0/1–3–1/0–1–4 quantities; in which the specific system is the weakest—intermediate—the most preferable. Based on the *FOM*, the distribution ranking shows uniform/Gaussian/adjusted (implying dye concentration) number density distribution order, so the most advantageous is the adjusted distribution, in accordance with the intuitive expectations.

Targets seeded with gold nanoprisms						
	Au-P	Au-SP-P	Au J-P	Au-A	Au-SP-A	Au-J-A
$\delta_{min\ PL}$	0.594	0.353	0.029	0.831	0.601	0.048
$t_{min\ PL}$ (fs)	499	288	250	361	500	240
$\Delta t_{min\ PL}$ (fs)	259	48	10	121	260	0
PL_{240fs} (J)	2.59×10^{-17}	6.82×10^{-18}	3.61×10^{-17}	1.89×10^{-17}	4.78×10^{-18}	2.77×10^{-17}
$\delta_{PL\ 240fs}$	0.724	0.407	0.051	0.946	0.674	0.048
FOM_{PL} (J)	3.58×10^{-17}	1.68×10^{-17}	7.14×10^{-16}	1.99×10^{-17}	7.02×10^{-18}	5.76×10^{-16}
$\delta_{min\ NFE}$	0.165	0.170	0.071	0.136	0.156	0.015
$t_{min\ NFE}$ (fs)	290	249	223	277	272	430
$\Delta t_{min\ NFE}$ (fs)	50	9	17	37	32	190
NFE_{240fs}	3.94	6.55	8.61	3.23	6.83	6.74
$\delta_{NFE\ 240fs}$	0.461	0.358	0.330	0.360	0.51	0.093
FOM_{NFE}	8.54	18.29	26.08	8.97	13.63	72.35
$\delta_{min\ E}$	0.590	0.349	0.029	0.821	0.588	0.048
$t_{min\ E}$ (fs)	500	285	250	361	500	240
$\Delta t_{min\ E}$ (fs)	260	45	10	121	260	0
E_{240fs} (Js)	1.69×10^{-31}	3.05×10^{-31}	2.35×10^{-31}	1.26×10^{-31}	2.12×10^{-31}	1.80×10^{-31}
$\delta_{E\ 240fs}$	0.721	0.401	0.049	0.940	0.664	0.048
FOM_E (Js)	2.35×10^{-31}	7.60×10^{-31}	4.79×10^{-30}	1.33×10^{-31}	3.20×10^{-31}	3.78×10^{-30}

Table S1. The minimal standard deviation (δ_{min_PL}, δ_{min_NFE}, δ_{min_E}), time instant (t_{min_PL}, t_{min_NFE}, t_{min_E}) and delay (Δt_{min_PL}, Δt_{min_NFE}, Δt_{min_E}) of it, the average value along the target (PL_{240fs}, NFE_{240fs}, E_{240fs}) and its standard deviation (δ_{PL_240fs}, δ_{NFE_240fs}, δ_{E_240fs}) at 240 fs and the *FOM* of the power-loss (FOM_{PL}), *NFE* (FOM_{NFE}) and deposited energy (FOM_E) in case of passive / active uniform (Au-P / Au-A), single-peaked Gaussian (Au-SP-P / Au-SP-A) and adjusted (Au-J-P / Au-J-A) nanoprism number density distribution. Color legend: black / grey is the weakest, blue / green is intermediate and red / orange is the most advantageous nanoprism number density distribution in passive / active targets. Green background indicates where the active targets are better than passive ones. All values carry a numerical uncertainty of ±1.5%, as determined from the mesh-convergence study.

$\delta_{min\ PL}$	$\delta_{min\ NFE}$	$\delta_{min\ E}$	$\Delta t_{min\ PL}$	$\Delta t_{min\ NFE}$	$\Delta t_{min\ E}$
Au-J-P	Au-J-A	Au-J-P	Au-J-A	Au-SP-P	Au-J-A
Au-J-A	Au-J-P	Au-J-A	Au-J-P	Au-J-P	Au-J-P
Au-SP-P	Au-A	Au-SP-P	Au-SP-P	Au-SP-A	Au-SP-P
Au-P	Au-SP-A	Au-SP-A	Au-A	Au-A	Au-A
Au-SP-A	Au-P	Au-P	Au-P	Au-P	Au-P
Au-A	Au-SP-P	Au-A	Au-SP-A	Au-J-A	Au-SP-A

Table S2. The ranking of the inspected targets in the minimal standard deviation and in its delay for the power-loss, *NFE* and the deposited energy in case of passive / active uniform (Au-P / Au-A), single-peaked Gaussian (Au-SP-P / Au-SP-A) and adjusted (Au-J-P / Au-J-A) gold nanoprism number density distribution. Color legend: black / grey is the weakest, blue / green is intermediate and red / orange is the most advantageous nanoprism number density distribution in passive / active targets. Green background indicates where the active targets are better than passive ones. All values carry a numerical uncertainty of ±1.5%, as determined from the mesh-convergence study.

PL_{240fs}	NFE_{240fs}	E_{240fs}	$\delta_{PL\ 240fs}$	$\delta_{NFE\ 240fs}$	$\delta_{E\ 240fs}$	FOM_{PL}	FOM_{NFE}	FOM_E
Au-J-P	Au-J-P	Au-SP-P	Au-J-A	Au-J-A	Au-J-A	Au-J-P	Au-J-A	Au-J-P
Au-J-A	Au-SP-A	Au-J-P	Au-J-P	Au-J-P	Au-J-P	Au-J-A	Au-J-P	Au-J-A
Au-P	Au-J-A	Au-SP-A	Au-SP-P	Au-SP-P	Au-SP-P	Au-P	Au-SP-P	Au-SP-P
Au-A	Au-SP-P	Au-J-A	Au-SP-A	Au-A	Au-SP-A	Au-A	Au-SP-A	Au-SP-A
Au-SP-P	Au-P	Au-P	Au-P	Au-P	Au-P	Au-SP-P	Au-A	Au-P
Au-SP-A	Au-A	Au-A	Au-A	Au-SP-A	Au-A	Au-SP-A	Au-P	Au-A

Table S3. The ranking of the inspected targets in the average value along the target and its standard deviation at 240 fs and the *FOM* of the power-loss, *NFE* and deposited energy in case of passive / active uniform (Au-P / Au-A), single-peaked Gaussian (Au-SP-P / Au-SP-A) and adjusted (Au-J-P / Au-J-A) gold nanoprism number density distribution. Color legend: black / grey is the weakest, blue / green is intermediate and red / orange is the most advantageous nanoprism number density distribution in passive / active targets. Green background indicates, where the active targets are better than passive ones. All values carry a numerical uncertainty of ±1.5%, as determined from the mesh-convergence study.

Mesh convergence study

A mesh convergence analysis was performed using continuously increasing mesh densities. The relative difference between the computed quantities (power-loss, deposited energy, and *NFE*) obtained with meshes not coarser or finer that 1.5-times the target mesh was below 1.5%, indicating that the model provides a converged numerical solution under the selected discretization settings (Figure S3). During constructing the mesh, the guideline was followed in accordance with the literature about that the spatial discretization in the dielectric regions should be scaled to approximately one-sixth of the wavelength, while in the vicinity of the gold nanoprisms the mesh was locally refined to ensure sufficient resolution of the high-gradient hot-spot regions characteristic of plasmonic near-fields (skin-depth/**5)**.

For the time-domain simulations, a time-step sufficiently small to resolve optical frequencies and the full spectral content of the femtosecond pulses was employed. The selected temporal discretization yielded stable and consistent convergence across the entire simulation time window and did not influence the determination of the standard deviations, *FOM* values, or the timing of the extrema.

Based on the combined spatial and temporal convergence study, the numerical model is stable, and the selected mesh and time-step parameters provide the accuracy required for the presented results.

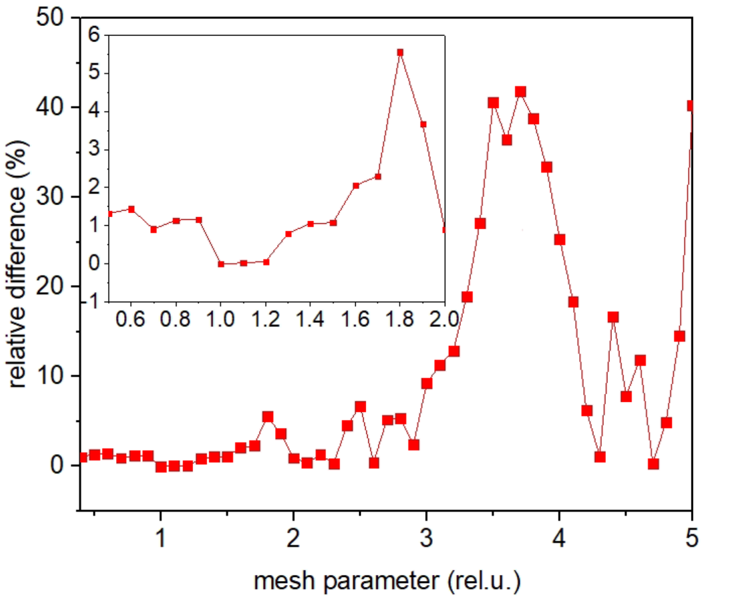

Figure S3. Mesh convergence study in a typical configuration. Mesh parameter is a global multiplicator, which means, that the mesh size ranged from 2-times smaller to 5-times larger, compared to the original mesh size. Inset: the main picture is zoomed in the relevant region.

Optical cross-section of individual nanoprisms

The wavelength dependent optical cross-section of the nanoprism proves well-defined resonance at the central wavelength of the laser pulse used for illumination (Figure S4). According to the average distance between the nanoprism in the target (>>100 nm) the near-field coupling between them is negligible, hence it can be extrapolated that the nanoprism distribution is resonant at the central wavelength as well.

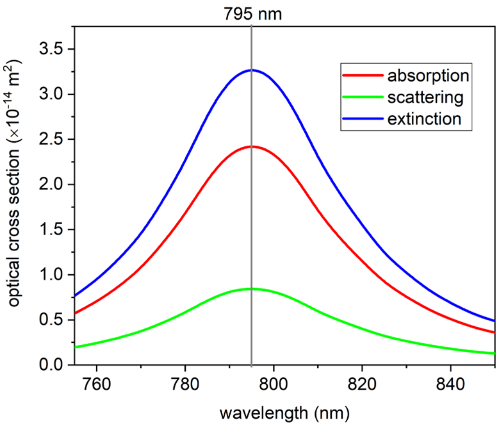

Figure S4 Absorption, scattering and extinction cross-section of the used nanotriangles.

Study of the optical properties of the dye media, absorption and near-field

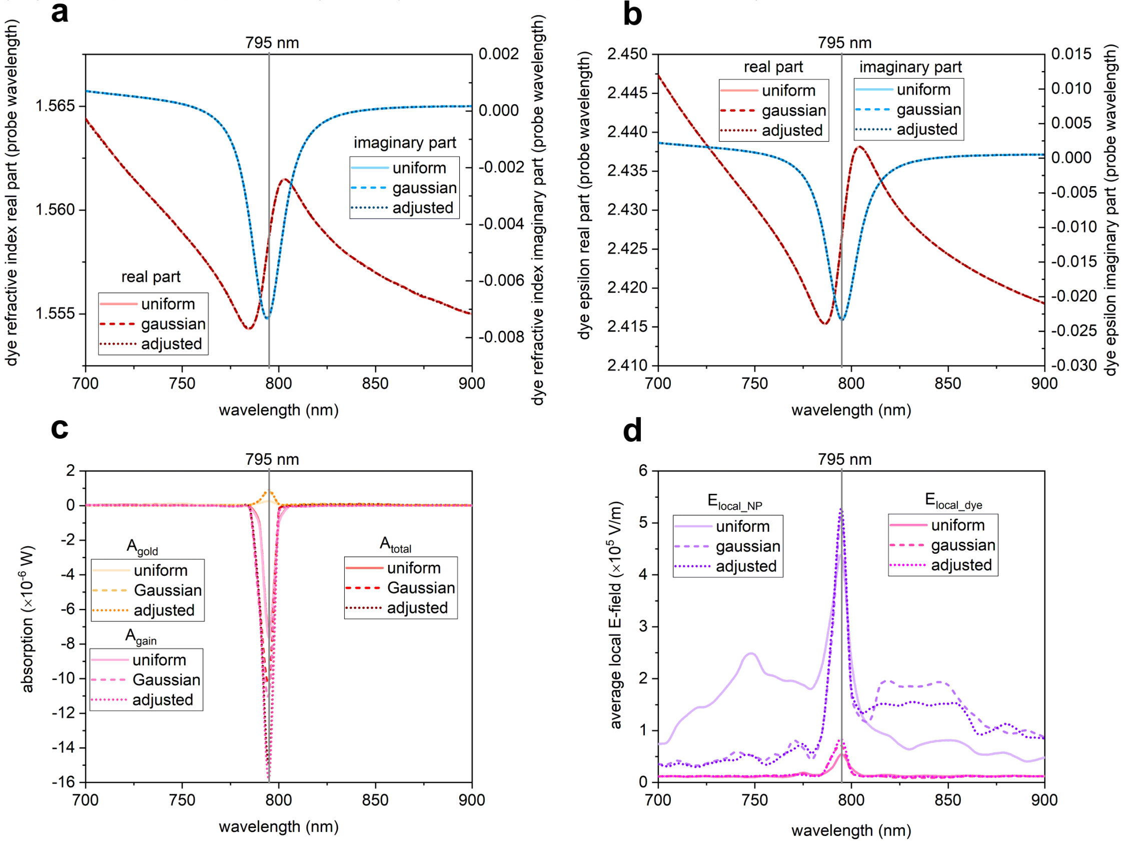

Figure S5 Spectra of the optical properties of the dye medium, absorptions and near-field enhancement. (**a**) Complex refractive index and (**b**) permittivity of the dye; (**c**) gold, gain and total absorption; and (**d**) average local **E**-field inside the gain medium and on the NPs' surface as the function of wavelength at E_{pump} = 2×10^{6} V/m and c=3.25×10^{26} m^{-3}.

The complex permittivity, index of refraction along with the absorption in the gold and gain medium and their sum, as well as the accompanying average local **E**-field on the surface of the nanotriangles and in the gain medium were inspected as a function wavelength. The real and imaginary part of the refractive index shows the characteristic oscillatory behavior in the special case of gain, i.e. with negative sign (using the convention of $e^{-i\omega t}$) around the emission wavelength of 795 nm (Figure S5a). The modulations are in the order of 0.01 (0.001) in the case of real (imaginary part) in this wavelength interval. The difference between the indexes in the case of different targets are minimal, which can be explained by that only a small fraction of the gain medium is around the randomly distributed nanoparticles, but it is averaged over a large volume. The complex permittivity spectra also show small modulations (Figure S5b). The real part of the permittivity is positive throughout the inspected interval, while the imaginary part is negative around 795 nm. The loss is significantly overcompensated at 795 nm, as the total absorption is negative for both nanoprism distribution (Figures S5c). The spectra of the average local **E**-field calculated on the surface of the nanoprisms is broad using uniform nanoprism number density distribution, and becomes narrower using Gaussian nanoprism number density distribution and adjusted number density and dye concentration distribution (Figure S5d). The largest near-field enhancement is achieved at 795 nm, but significant near-field enhancement can be also achieved at smaller / larger wavelength using uniform / Gaussian and adjusted number density distribution. The smallest / intermediate / largest gain and *NFE* can be achieved in uniform / Gaussian / adjusted distribution.
